# Reducing Methylglyoxal Rescues Obesity Related Phenotypes and Lifespan in a Leptin Receptor Deficient Mouse Model

**DOI:** 10.1093/geroni/igab046.2563

**Published:** 2021-12-17

**Authors:** Lauren Wimer, Martin Valdearcos Contreras, Muniesh Muthaiyan Shanmugam, Jessica Ramirez, Jennifer Beck, Suneil Koliwad, Pankaj Kapahi

**Affiliations:** 1 Buck Institute for Research on Aging, Novato, California, United States; 2 UCSF Diabetes Center, UCSF Diabetes Center, California, United States; 3 UCSF Diabetes Center, 94143, California, United States

## Abstract

Obesity remains one of the leading risk factors for aging and age-related diseases such as Alzheimer’s and type II diabetes, and effects 40% of the US population. The occurrence of obesity has been closely tied to an increase in sugar consumption with chronic hyperglycemia enhancing glycolysis. One of the byproducts of glycolysis is methylglyoxal (MGO), a reactive precursor for advanced glycation end-products (AGEs), which drives type II diabetes and its complications. We hypothesize that an MGO-derived AGE, MG-H1, affects hypothalamic regulation of food consumption and metabolism parallel to the leptin pathway in mice fed a high carbohydrate diet. Exogenous supplementation of MG-H1 increased food consumption rates and weight gain. Conversely, glycation byproduct lowering compounds (GLY-LOW), a customized chemical cocktail that blocks the production of MGO, rescued over-feeding phenotypes in wild-type mice. Furthermore, GLY-LOW treatment in a leptin receptor deficient mouse model rescued weight gain, diabetic phenotypes and lifespan. RNA sequencing of the hypothalamus of leptin receptor deficient mice treated with GLY-LOW showed significant downregulation of Rax, a gene responsible for tanycyte differentiation, and several genes involved in feeding and aging. We propose that specific cells in the hypothalamus both make and respond to MG-H1. We will also discuss evidence for the potential of GLY-LOW as a new class of therapeutics that reduce the effects of glycation to reduce food intake and slow aging.

